# Understanding the Molecular Mechanism of Vestibular Schwannoma for Hearing Preservation Surgery: Otologists’ Perspective from Bedside to Bench

**DOI:** 10.3390/diagnostics12051044

**Published:** 2022-04-21

**Authors:** Makoto Hosoya, Takeshi Wakabayashi, Koichiro Wasano, Takanori Nishiyama, Nobuyoshi Tsuzuki, Naoki Oishi

**Affiliations:** 1 Department of Otolaryngology-Head and Neck Surgery, Keio University School of Medicine, 35 Shinanomachi, Shinjuku-ku, Tokyo 160-8582, Japan; mhosoya1985@gmail.com (M.H.); t12wakabayashi@yahoo.co.jp (T.W.); tnmailster@gmail.com (T.N.); n.tsuzuki@keio.jp (N.T.); 2 Department of Otolaryngology and Head and Neck Surgery, Tokai University School of Medicine, 143 Shimokasuya, Isehara 259-1193, Kanagawa, Japan; wasano@a5.keio.jp

**Keywords:** vestibular schwannoma, hearing loss, hearing preservation surgery, *NF2* gene

## Abstract

Vestibular schwannoma is a clinically benign schwannoma that arises from the vestibulocochlear nerve that causes sensorineural hearing loss. This tumor is clinically and oncologically regarded as a benign tumor as it does not metastasize or invade surrounding tissues. Despite being a benign tumor, its management is difficult and controversial due to the potential serious complications, such as irreversible sensorineural hearing loss, of current interventions. Therefore, preventing hearing loss due to the natural course of the disease and complications of surgery is a challenging issue for an otologist. Improvements have been reported recently in the treatment of vestibular schwannomas. These include advances in intraoperative monitoring systems for vestibular schwannoma surgery where the risk of hearing loss as a complication is decreased. Precise genomic analysis of the tumor would be helpful in determining the characteristics of the tumor for each patient, leading to a better hearing prognosis. These procedures are expected to help improve the treatment of vestibular schwannomas. This review summarizes recent advances in vestibular schwannoma management and treatment, especially in hearing preservation. In addition, recent advances in the understanding of the molecular mechanisms underlying vestibular schwannomas and how these advances can be applied in clinical practice are outlined and discussed, respectively. Moreover, the future directions from the bedside to the bench side are presented from the perspective of otologists.

## 1. Introduction

Vestibular schwannoma is a clinically benign schwannoma that arises from the vestibulocochlear nerve (VIIIth cranial nerve) that runs through the internal auditory canal. The vestibulocochlear nerve comprises of the cochlear nerve, the superior vestibular nerve, and the inferior vestibular nerve. Most vestibular schwannomas originate from the inferior vestibular nerves between the other two nerves [1,2]. Vestibular schwannoma can arise anywhere along the course of the vestibular nerve, running from the cistern to inside the vestibular organ. However, the vast majority of vestibular schwannomas are observed from the cistern portion to the porus of the inner ear canal. Therefore, typical vestibular schwannomas present as cerebellopontine angle tumors. Vestibular schwannoma accounts for about 8–10% of intracranial tumors and almost 80% of cerebellopontine angle tumors [3].

The incidence of vestibular schwannoma is estimated to be approximately 20 new cases per million people each year [4] with a lifetime prevalence of approximately one case in 500 persons [5]. Vestibular schwannoma can cause various symptoms, including dizziness due to its effect on the vestibular nerve, and sensorineural hearing loss due to damage to the cochlear nerves. These damages lead to hearing loss caused by the secreted molecules of the schwannoma [6,7] or the schwannoma-associated inflammatory response [8], as well as direct compression of the tumor and inner ear dysfunction [9]. It can also cause facial paralysis due to compression of the facial nerve, which runs parallel to the vestibulocochlear nerve in the inner ear canal. In addition, as the tumor grows, it can cause pressure on the brain stem, which can be fatal.

Vestibular schwannomas are clinically and oncologically regarded as benign tumors. Despite this, the management of vestibular schwannoma is difficult and controversial because vestibular schwannoma interventions can cause complications. Considering the interventions for vestibular schwannomas, severe complications, including irreversible sensorineural hearing loss and facial nerve paralysis, must be considered. Therefore, while vestibular schwannoma is a benign tumor, when and how to treat it is not an easily solved clinical question and highly depends on several factors and conditions.

Improving vestibular schwannoma management to prevent complications such as hearing loss and facial nerve palsy is desirable. In particular, from the viewpoint of an otologist, hearing preservation from the vestibular schwannoma is an essential but challenging subject, as well as preventing facial nerve paralysis. With recent advances in surgical equipment systems and the molecular understanding of the disease, vestibular schwannoma treatment has changed and improved. This review summarizes the recent advances in vestibular schwannoma management and treatment. Future directions from bedside to bench will be shown from the viewpoint of the otologist.

## 2. The Natural Course of Hearing Levels in Vestibular Schwannoma Patients

Hearing loss caused by vestibular schwannoma is not always severe or permanent. This hearing loss can be mild to moderate [10,11,12,13] and reversible in several patients [14,15]. An estimated 10–30% of patients diagnosed with vestibular schwannoma develop sudden sensorineural hearing loss [16,17,18] and 3–5% of patients diagnosed with sudden sensorineural hearing loss exhibit vestibular schwannomas [19,20]. This hearing loss caused by sporadic vestibular schwannomas is often treated with corticosteroids according to the treatment protocols used for idiopathic sudden sensorineural hearing loss. Several earlier reports have shown that hearing recovers with corticosteroid treatment [21,22,23]. This heterogeneity of hearing loss caused by vestibular schwannoma makes decision making in terms of when interventions should be carried out difficult.

Thus far, Stangerup et al. reported that, while 53% of patients had good hearing and speech discrimination upon diagnosis, this became 31% after ten years of observation [24]. Recently, Wasano et al. reported that the recovery rate of vestibular schwannoma-associated sudden sensorineural hearing loss decreases with increasing episodes of hearing loss [14]. They also estimated that the recurrence rate of hearing loss within one year was 25%. This suggests that patients with vestibular schwannoma who apparently recovered from hearing loss should be considered to undergo further surgical interventions due to a high risk of hearing loss recurrence.

## 3. Importance of Hearing Preservation after Vestibular Schwannoma Surgery

**Figure 1:**
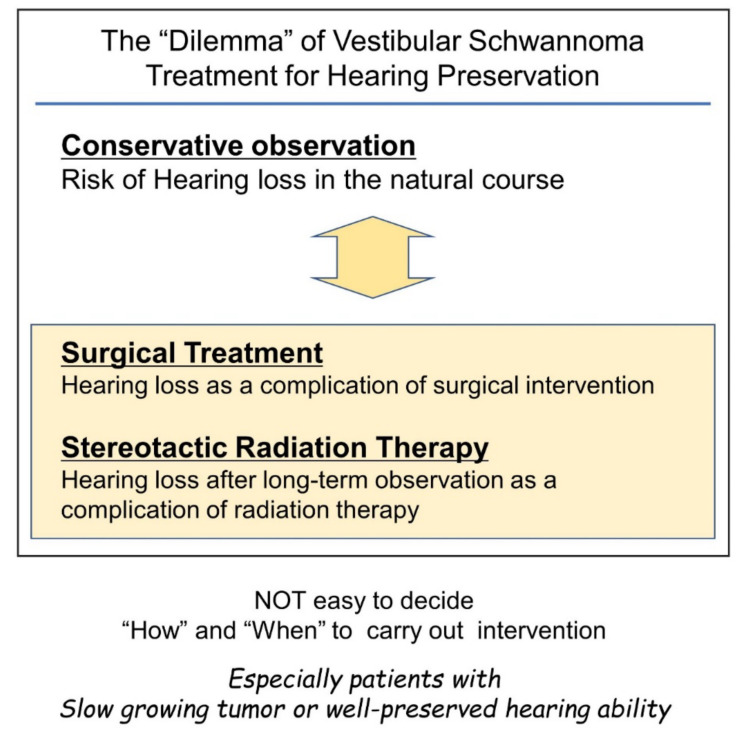
Difficulties in timing considerations in vestibular schwannoma intervention with the goal of preserving hearing. Conservative observation and intervention with surgery or stereotactic radiotherapy can cause hearing loss. Therefore, the decision to intervene can be a difficult decision for the surgeon.

Considering the risk of gradual or sudden hearing loss in the natural course of disease for patients with vestibular schwannoma [14,25], hearing preservation through the surgical removal of the tumor would be ideal. However, surgical intervention itself is a risk factor for hearing loss, and there is no ideal surgical approach to ensure hearing preservation. Hence, surgeons are faced with a dilemma (Figure 1).

Therefore, if the tumor size is not life-threatening, non-hearing preservation surgery can be chosen once hearing loss sets in by natural course. This type of surgical decision making has been widely applied. However, the importance of hearing preservation has been underestimated in this context.

Preserving residual hearing in vestibular schwannoma patients is necessary for several reasons. Hearing loss on the affected side due to either the natural course of the disease or the side effects of the interventions usually results in impaired sound localization and difficulty in understanding speech in the presence of background noise [26,27]. However, if serviceable hearing can be spared, hearing aid use can be considered. Recently, Kitamura et al. reported the possible importance of residual hearing after hearing preservation surgery for the treatment of tinnitus [28]. It has also been reported that tinnitus negatively affects the quality of life of patients with vestibular schwannomas [29,30,31]. These previous studies suggest that hearing preservation is desirable in tinnitus management and it improves the patients’ quality of life.

## 4. Interventions for the Vestibular Schwannoma

There are several management methods for vestibular schwannomas. A “wait-and-scan” policy, in which patients’ tumors are observed once or twice by MRI per year, is well applied to patients with non-growing tumors [32,33,34,35]. Typically, tumors smaller than 1.5 cm in diameter located on the cerebellopontine angle are considered for a wait-and-scan approach. Once tumor growth has been detected with or without cystic changes, interventions should be considered, and, especially in patients who are candidates for hearing preservation, surgery should be considered [36]. Because of the potential side effects of treatments and the fact that the tumor is benign and non-invasive, in several cases, such as elderly patients who do not want surgery, or patients with very slow-growing tumors, the “wait-and-scan” policy may still be preferable.

There are several interventions available for a growing vestibular schwannoma, including radiotherapy and surgical resection. Stereotactic radiosurgery, such as Gamma-Knife [37,38], has been widely used in radiation therapy for vestibular schwannoma. Stereotactic radiosurgery aims to prevent tumor growth but without expectation of a radiological cure, similar to other radiation therapies for malignant tumors. Stereotactic radiosurgery showed a high long-term local control rate [39] and had merit that it can be applied for the relatively large tumor that cannot be surgically removed [40]. Stereotactic radiosurgery is an effective treatment option for small- to medium-sized (<3 cm) vestibular schwannomas without significant mass effect, while large tumors causing brainstem compression or symptoms of mass effect require surgical intervention. Stereotactic radiosurgery has similar local control rates compared to surgery in appropriately selected patients, typically those without significant mass effect or brain compression [41]. In some instances, stereotactic radiosurgery provides better functional outcomes [41].

Although stereotactic radiosurgery has several merits and is widely used, it still has the disadvantage of hearing loss. It is believed that stereotactic radiosurgery has a high hearing preservation rate. However, recent long-term observations showed that patients’ hearing after stereotactic radiosurgery gradually decreased [42,43,44,45], and serviceable hearing levels are lost in most patients in a span of ten years [43]. This result suggests that stereotactic radiosurgery can only achieve temporary hearing preservation; therefore, this intervention may not be suitable for younger patients.

Neurofibromatosis type II is often treated by a neurosurgeon and less often by an otolaryngologist, requiring a different treatment plan. In neurofibromatosis type II, special consideration should be given to the possibility of bilateral hearing loss due to bilateral vestibular schwannomas. Treatment strategies for sporadic vestibular schwannomas rarely apply to neurofibromatosis type II cases, as contralateral hearing levels and the possible presence of other brain tumors such as meningiomas affect the treatment strategy for neurofibromatosis type II.

## 5. Surgical Approach for the Vestibular Schwannoma

Surgical resection can be performed for all tumor sizes. Usually, large tumors, such as those associated with brainstem compression, are resected by neurosurgeons, and otologists treat relatively smaller tumors that are limited to the inner ear canal or those that slightly reach the brainstem. The middle fossa approach, translabyrinthine approach, and retrosigmoid approach are thought to be the three primary microsurgical approaches; however, other approaches are also used to remove vestibular schwannomas, each with benefits and limitations.

Surgical interventions for vestibular schwannoma can be categorized into two types depending on the expected postoperative hearing results: non-hearing preservation surgery and hearing preservation surgery. Most non-hearing preservation surgeries are performed using the translabyrinthine approach [46]. The transotic approach can also be used [47,48]. Recently, the exclusive endoscopic transcanal transpromontorial approach has been reported as a non-hearing preservation surgery [49,50]. Several surgical methods can be used for hearing preservation, including the retrosigmoid approach, the middle temporal fossa approach, and the retrolabyrinthine approach. Recently, the retrolabyrinthine meatotomy technique combined with the retrosigmoid approach has been reported [51].

While the surgical approach can be easily divided into two groups, it is difficult to define “what is good and serviceable hearing” or “what is hearing worth to be preserved”. Therefore, several criteria have been used. Historically, Wade and House described the “50/50 rule”, which defined serviceable hearing as a pure tone average (PTA) of ≤50 dB with a speech discrimination score (SDS) of 50% or better [52]. In 1988, the Gardner–Robertson Scale was developed, in which Grade I (Good: PTA ≤ 30 dB with SDS ≥ 70%) and Grade II (Serviceable: PTA of 30–50 dB with SDS ≥ 50%) are considered “useful hearing” [53]. In 1995, the American Academy of Otolaryngology Head and Neck Surgery guideline was established for reporting hearing outcomes after hearing preservation surgery for lateral skull base surgery, in which all patients with SDS < 50% are considered as having non-serviceable hearing [54]. In 2003, the Tokyo classification was developed [55]. These criteria help report the results of surgical interventions and are usually also used for selecting the candidate for hearing preservation surgery.

Based on these previous criteria, candidates for hearing preservation surgery have often been defined as patients with PTA ≤ 30 dB with SDS ≥ 70% [56,57] or with PTA of 30–50 dB with SDS ≥ 50% [58,59]. However, as mentioned above, it was suggested that any level of residual hearing might be helpful for tinnitus control or the improvement of patients’ quality of life. Moreover, the residual hearing, even if it was at an “unserviceable level”, might broaden the possibility of hearing rehabilitation after vestibular schwannoma surgery [27]. In our department, we have not uniformly selected the candidate for the hearing preservation surgery by the preoperative hearing level; however, our previous study indicated that patients with shorter auditory brainstem response (ABR) wave V latency or showing a higher otoacoustic emission (OAE) response are the best candidates for the proposed hearing preservation surgery [60]. Therefore, we believe that the surgeon should consider operating at an early stage before the ABR and OAE responses become disturbed.

The selection of the surgical approach is dependent on the degree of residual hearing, tumor size, tumor location, and whether hearing preservation is preferred. If hearing preservation surgery is selected, the central focus of the procedure is the preservation of auditory function. However, irrespective of which surgical category is selected, the surgeon must attempt to prevent facial nerve palsy, particularly in cases with no evident preoperative deficit. Intraoperative neural monitoring is a widely used method to facilitate postoperative hearing and prevent facial nerve function deficits.

## 6. Intraoperative Electrophysiological Monitoring for Vestibular Schwannoma Surgery

Since the first report of intraoperative ABR monitoring in vestibular schwannoma surgery in 1982 [61], it has become a standard monitoring method, and its clinical value is now broadly accepted in the field. Since then, other modalities have been introduced into clinical practice, including direct eighth cranial nerve monitoring via cochlear nerve action potentials (CNAP) [62,63,64,65] or dorsal nucleus action potential (DNAP) [66,67,68], electrocochleography [69,70], and OAE [71].

However, intraoperative ABR monitoring has two major limitations. First, ABR detection is time-consuming. ABR measurement requires an average of 500–2000 stimulations and a duration of more than one minute. Although this time period is acceptable when applying ABR as a hearing test in an outpatient setting, it is problematic in intraoperative monitoring. Second, vestibular schwannomas impede acoustic neuron conduction. As a result, the amplitude of the ABR decreases and, in some cases, intraoperative ABR waveforms cannot be obtained. In the 2010s, a novel form of intraoperative monitoring for hearing function was developed, i.e., DNAP monitoring [72]. In DNAP monitoring, the detection probe is set on the dorsal cochlear nucleus of the brainstem. The action potential of the dorsal cochlear nucleus is detected using this probe. DNAP has high sensitivity in detecting electrical signals and can detect signals that ABR overlooks. Moreover, the DNAP system requires an average of only 100–200 stimulations. Intraoperative DNAP signals can thus be obtained every 10 s. In contrast, when using the DNAP, putting the detection probe on the brainstem is required, which is not needed for ABR monitoring. Therefore, DNAP monitoring cannot be applicable in the middle fossa approach, although ABR monitoring can be used in all hearing preservation surgeries.

Electromyographic monitoring systems, such as the Medtronic NIM system, are widely used [73,74,75,76]. However, this system involves the detection of sporadic firing of the facial nerve via intrinsic physiological stimulation. Thus, this system cannot be used for continuous intraoperative monitoring. Advanced monitoring systems with continuous monitoring have been developed to overcome this limitation [72,77,78]. The monitoring system using facial nerve root exit zone-elicited compound muscle action potential (FREMAP) is one such system that has been recently introduced. Although the FREMAP monitoring system needs to set an electric stimulation probe to the main trunk of the facial nerve, this probe enables continuous intraoperative stimulation of the facial nerve, followed by detection of a facial muscle action potential. FREMAP monitoring enables stimulation of the facial nerve and the detection of facial muscle action potentials to be performed continuously throughout the surgical procedure [79]. Thus, the surgeon can obtain continuous quantitative data concerning facial nerve status during tumor resection. In addition, the surgeon can abandon the procedure or select an alternative resection route, if necessary. While the electrode for FREMAP monitoring is designed to be put on the main trunk of the facial nerve in the surgical view of the retrolabyrinthine approach or the retrosigmoid approach, it could be applied to other approaches, including non-hearing preservation surgeries.

## 7. Retrolabyrinthine Approach under Reinforced Continuous Intraoperative Monitoring with FREMAP and DNAP

The retrolabyrinthine approach is a surgical approach for vestibular schwannomas that can preserve patients’ hearing [60,80,81,82,83,84]. In the retrolabyrinthine approach, the tumors are approached through the area posterior to the posterior semicircular canal and prior to the sigmoid sinus [83]. This approach has several advantages compared with other approaches that can preserve hearing. The first advantage is that this approach does not require craniotomy, which is required by the retrosigmoid and middle fossa approaches. Therefore, less pressure is applied to the temporal lobe or cerebellum. The second advantage of the retrolabyrinthine approach is that the risk of encountering the facial nerve first in the inner ear canal is relatively lower compared with the middle fossa approach. The third advantage of this approach is that DNAP and FREMAP electrodes, which cannot be used in the middle fossa approach, can be used because the brain stem is visible.

However, there are several disadvantages of the retrolabyrinthine approach as well. First is that this approach cannot be applied to some patients because of certain anatomical features of the temporal bones. Patients with one or more of the following anatomical features may be poor candidates for this approach: high jugular bulb, less developed bony cells posterior to the labyrinth, less developed mastoid air cells, and less developed sigmoid sinus on the opposite side of the surgical site. The second disadvantage of the retrolabyrinthine approach is that the surgical field is relatively narrower than other approaches, and the fundus of the inner ear canal cannot be visualized by surgical microscopy alone. Although the use of the endoscope can overcome this limitation to some extent [85,86,87], this approach is more suitable for smaller tumors (Koos classification I or II) with tumor-free space on the fundus of the inner ear canal.

**Figure 2:**
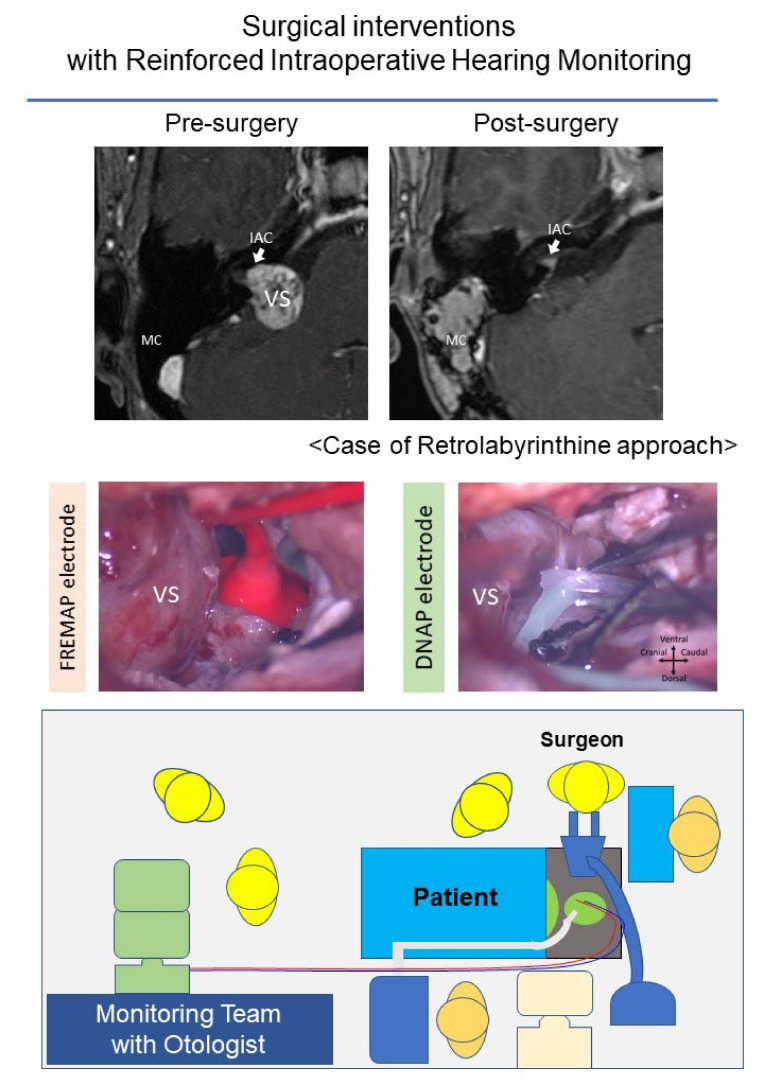
An example of the operation settings of vestibular schwannoma surgery with continuous monitoring (case of right-sided vestibular schwannoma). Combining intraoperative continuous monitoring systems with one of the hearing preservation surgeries is an effective approach to reduce the risk of hearing loss. This figure shows an example of a combination of the retrolabyrinthine approach and the FREMAP/DNAP monitoring system. After opening the dura in the cisternal portion, electrodes of FREMAP and DNAP are placed to monitor facial nerve function and hearing. If any changes in the monitoring are detected, caution should be noted by the team, which usually includes an otologist. These cautions are helpful for surgeons to avoid unintended damage to the nerves. VS: vestibular schwannoma, IAC: internal auditory canal, MC: mastoid cavity.

Previously, Bento et al. reported hearing preservation in 31.8% of retrolabyrinthine approach cases, all of whom presented with a minimum AAO-HNS level of Class B [88]. Recently, we established a retrolabyrinthine approach with reinforced continuous intraoperative FREMAP and DNAP monitoring [60,79] (Figure 2). A combined retrolabyrinthine and reinforced continuous intraoperative monitoring approach is comparable to the middle fossa and retrosigmoid approaches, which are associated with hearing preservation rates of 40–85% [60]. Under this novel approach, combined with appropriate patient selections through preoperative evaluation with ABR and OAE, 50–100% hearing preservation can be achieved postoperatively. Excellent facial function was achieved in 100% of patients [60]. These results suggest that in small growing tumors, surgical intervention with adequate monitoring systems should be considered before residual hearing worsens.

## 8. From Bedside to Bench: The Importance of Investigating the Molecular Mechanism of Vestibular Schwannoma for Hearing Loss Prevention

As discussed above, the hearing preservation rate has recently improved in patients with vestibular schwannoma. Therefore, there has been an increase in the number of patients with vestibular schwannoma who have undergone hearing preservation surgery. In particular, hearing preservation surgery has increased in younger patients where long-term postradiotherapy hearing loss would be a problem, or patients with normal hearing and smaller tumors who tend to avoid surgery due to the risk of postoperative hearing loss.

Under such clinical situations, the precise prediction of tumor growth or the risk of hearing loss is required to determine the optimal timing of intervention. Moreover, the prediction of long-term prognosis after hearing preservation surgery is critical. However, a method to predict the clinical course of each patient has not been established. Recently, especially in the study of malignant tumors, the molecular biological investigation of surgically removed tumors from each patient has been thought of as being a feasible way to predict the clinical course of the patient. This approach has been broadly used in clinical settings for other tumors. For example, the investigation of *BRAF* gene variants in lung cancer and melanoma has been used to predict its clinical features and choices of treatment [89,90].

To date, several previous reports have suggested the possible use of these molecular biological approaches for vestibular schwannoma cases [91,92,93]. Moreover, it would be desirable to investigate each patient’s resected tumor during hearing preservation surgery to predict long-term prognosis. However, this method has not yet been established. In the following sections, we will review the possibility and utility of investigating the molecular characteristics of vestibular schwannoma to prevent hearing loss and discuss future directions.

## 9. Clinicians Needs to Understand the Molecular Mechanisms Underlying Vestibular Schwannoma Tumorigenesis

Clinical otologic surgeons await the unveiling of the molecular mechanisms underlying the tumorigenesis of vestibular schwannoma, which can be used to develop novel prognosis prediction methods based on somatic mutations of the tumors, for several reasons.

First, this new prediction method would be helpful in postoperative management. If in the future, after hearing preservation, genomic testing of the resected tumor reveals a high probability of tumor regrowth surgery, radiation therapy for salvage can be considered early. In contrast, if the resected tumor is found to have a lower growth rate through somatic gene investigation, the postoperative management of the tumor will be easier, and even if a small residual tumor is detected, the surgeon can reassure the patient.

Second, it would broaden the indications for surgery and possible surgical approaches. If the prediction of tumor growth or the sensorineural hearing loss risk is possible, a vestibular schwannoma biopsy can be considered along with complete tumor resection, especially under the reinforced monitoring of the facial nerve and hearing. Moreover, if rapid intraoperative diagnosis for these risks is possible, the results will help surgeons decide whether to focus on tumor resection or hearing preservation. In the case of a low-risk tumor, preservation of the hearing can be prioritized, and in the case of a high-risk tumor, the total resection of the tumor can be prioritized. In this context, the surgery starts with hearing preservation, such as via the retrolabyrinthine approach, and then, depending on the result of the rapid diagnosis made through intraoperative biopsy, the surgeon can convert to a non-hearing-preservation approach, such as the translabyrinthine approach, if total resection of the tumor is preferable. In such cases, concurrent cochlear implantation [94,95,96,97,98] or auditory brainstem implantation [99,100] is worth considering.

Third, understanding the molecular mechanisms underlying the development of sporadic vestibular schwannoma may assist in developing new drug therapies. Neurological disorders such as sensorineural hearing loss caused by vestibular schwannoma usually result from tumor compression and impaired blood flow. The mechanism of neurological damage, however, has not yet been clarified, making it difficult to predict the progression of hearing loss or sudden hearing loss caused by vestibular schwannoma. However, novel therapies can act on this underlying molecular mechanism. Recently, several candidate drugs for the treatment of neurofibromatosis type II have been reported [101,102,103,104]. In such cases, the risk of sensorineural hearing loss could be decreased, and patients could be saved from hearing loss.

## 10. Molecular Biology of Vestibular Schwannoma

**Figure 3:**
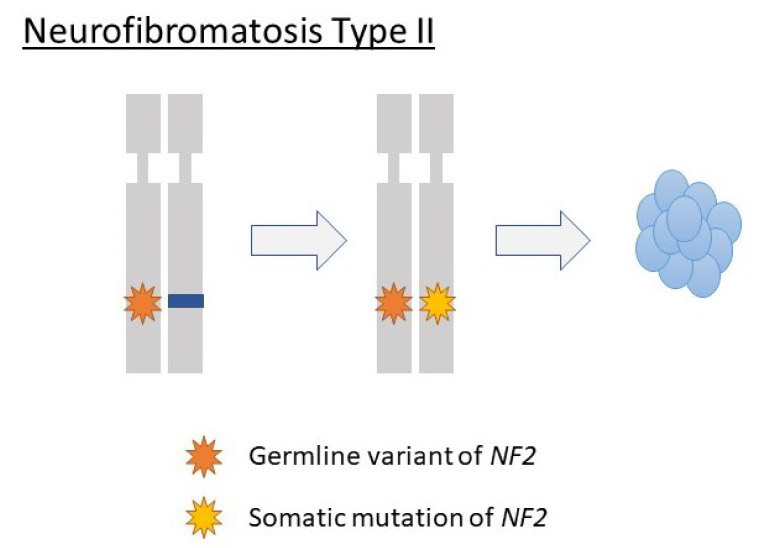
Molecular mechanism of tumorigenesis in vestibular schwannoma caused by neurofibromatosis type II. In neurofibromatosis type II, both germline variants and somatic mutations of the *NF2* gene are associated with the development of vestibular schwannoma.

Much of the knowledge on the molecular biology of vestibular schwannoma has been obtained from patients with neurofibromatosis type II, an autosomal dominant hereditary disease that manifests with bilateral vestibular schwannomas. Previous investigations have revealed that germline variants of the *NF2* gene, a tumor suppressor gene located at 22q12 [105], can cause vestibular schwannoma [106,107,108,109]. *NF2* encodes Merlin, a cytoskeletal protein [107]. Variants in both alleles of this gene trigger tumorigenesis in vestibular schwannoma. In cases of neurofibromatosis type II, the germline variant of the congenital *NF2* gene, combined with the acquired inactivation of this gene in somatic cells, leads to vestibular schwannomas, similar to the well-known “two-hit” theory for tumors caused by the inactivation of other tumor suppressor genes [110] (Figure 3).

Unlike neurofibromatosis type II cases, sporadic unilateral vestibular schwannoma cases do not involve germline variants, except in schwannomatosis cases [111], and somatic genomic changes are thought to cause these tumors. These somatic genomic changes have been thought to influence the characteristics of the tumor, including growth speed, cystic changes, effects on hearing levels, and tumorigenesis [112,113,114]. However, these somatic genetic changes in solitary vestibular schwannoma cases are not fully understood. In particular, the relationship between hearing loss caused by sporadic vestibular schwannoma and somatic mutations has not been elucidated.

It has been reported that somatic alterations in the *NF2* gene are critical factors in the pathogenesis of sporadic vestibular schwannomas [115,116,117]. It has been revealed that somatic mutations in both alleles of this gene trigger tumorigenesis in vestibular schwannoma [114,118]. Additionally, the combination of *NF2* gene mutations and loss of heterozygosity (LOH) caused by partial or complete loss of chromosome 22, where the *NF2* gene is located, can lead to sporadic vestibular schwannoma. Previously, it has been reported that there is at least one allele of the *NF2* gene mutation in 50–85% cases [119,120,121,122,123,124,125,126,127,128,129], and a double hit of the *NF2* gene has been reported in 30–62% cases [121,122,125,126,127,128,129,130]. Recently, Carlson et al. used whole-exome sequencing, mate-pair analysis, and RNA-seq to investigate *NF2* genes in sporadic vestibular schwannomas. They revealed “two-hit” alterations in the *NF2* gene in every tumor [114]. This variety of the reported percentage in previous reports about the incidence of *NF2* gene mutations might indicate that it may vary depending on the detection method and patient population. However, the mutation or inactivation of the *NF2* gene is important for the tumorigenesis of sporadic vestibular schwannoma. Further investigation is required in future studies.

**Figure 4:**
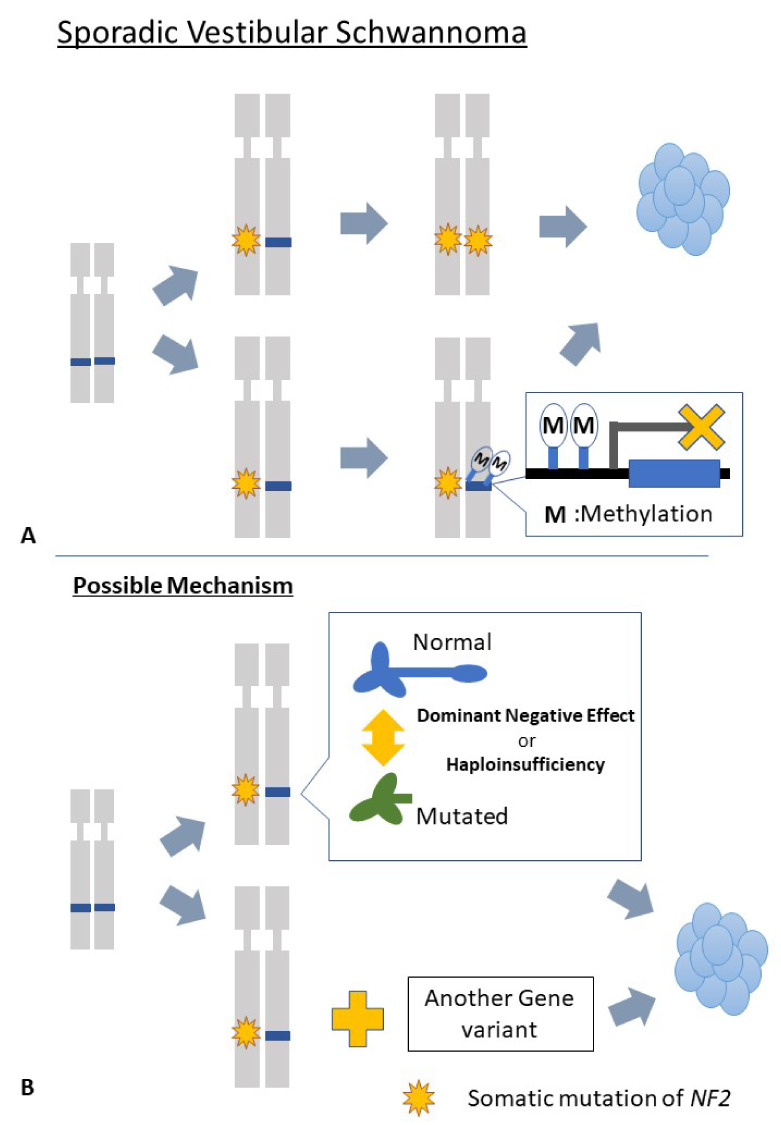
Molecular mechanism of the tumorigenesis in sporadic vestibular schwannoma. In sporadic vestibular schwannoma, somatic mutation of the *NF2* gene is essential for tumor development, but the mechanism has not been fully understood in contrast to neurofibromatosis cases. In many cases, biallelic *NF2* gene variants lead to tumorigenesis (**A**); however, other possible mechanisms have been suggested (**B**).

The “two-hit” model of the *NF2* genes can explain many of the sporadic vestibular schwannoma cases. However, in a certain percentage of sporadic vestibular schwannoma cases, no mutations in the *NF2* gene or mutations in only one allele were detected (Figure 4A). The molecular mechanisms underlying tumorigenesis need to be investigated in these cases. Epigenetic modification of *NF2* genes is one of the possible mechanisms of *NF2* gene inactivation in sporadic vestibular schwannomas. Epigenetic modification with methylation leads to the inhibition of gene transcription, and methylation-dependent silencing of the *NF2* gene has been reported [131,132]. However, several previous reports have suggested that promoter methylation is an uncommon mechanism of *NF2* inactivation in sporadic vestibular schwannomas [133,134]. Thus, the effect of epigenetic modification of the *NF2* gene on the pathogenesis of sporadic vestibular schwannomas is controversial.

There are several other possible mechanisms that cause tumorigenesis in sporadic vestibular schwannoma (Figure 4B). Tumorigenesis caused by a monoallelic mutation with a dominant-negative effect is a possible mechanism. In these situations, even if functional Merlin could be produced, a specific type of mutated Merlin would inhibit the normal function of the normal Merlin, leading to tumorigenesis. A single mutation that causes haploinsufficiency, with or without mutations in other genes, may also be one possible mechanism. Chen et al. reported that monoallelic mutation of the *NF2* gene could lead to the development of a slow-growing sporadic vestibular schwannoma observed in elderly patients [129]. Another possible mechanism is that unidentified non-*NF2* gene mutations may affect transcription of the remaining normal *NF2* and act as driver mutations.

While no specific gene has been identified other than the *NF2* gene, several other possible mechanisms of tumorigenesis or tumor growth have been revealed [135,136,137,138], especially in the recent comprehensive data provided by omics analysis [139,140,141,142,143,144]. For example, Seo et al. reported that apoptosis is associated in a complex manner with the pathophysiology of vestibular schwannoma [140]. Recently, Ren et al. reported that the abundance of proteolytic activity of matrix metalloprotease 14 in the plasma and tumor secretions of vestibular schwannoma patients correlated with clinical parameters and the degree of sensorineural hearing loss [145]. Other genes could be involved not in tumorigenesis but in the growth of the vestibular schwannoma, as observed in glioblastoma [146,147]. These attempts to determine the molecular biological mechanisms of vestibular schwannomas may be another feasible approach that should be explored in future research.

## 11. Possibility of Intraoperative Rapid Genomic Test or Liquid Biopsy

**Figure 5:**
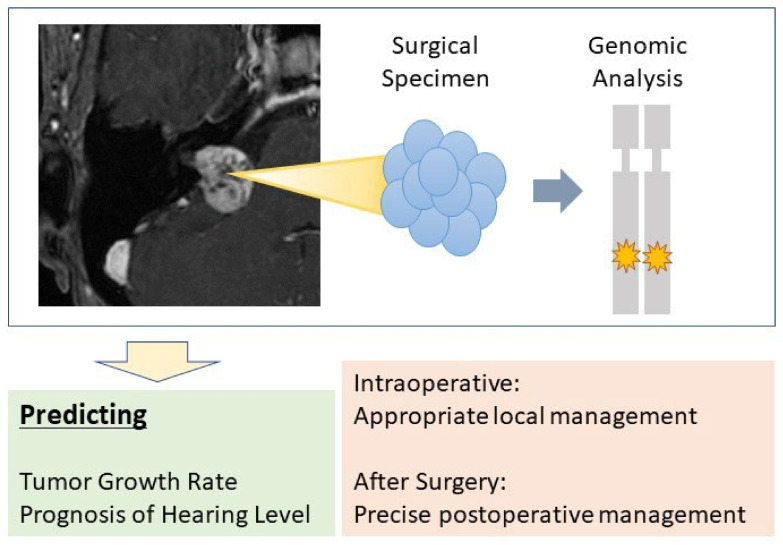
Clinical importance of genomic analysis for vestibular schwannoma. Predicting the clinical prognosis based on genomic analysis obtained from surgical specimens will help improve postoperative management. If intraoperative rapid analysis is possible, it will also help to improve the quality of the surgery by achieving the appropriate local management.

To date, most of the genomic test results of tumors are available several days after surgery. However, recent advances in technology have made intraoperative rapid genomic testing possible in some cases. For example, in glioma cases, intraoperative molecular characterization is available [148]. If this intraoperative genomic test, such as targeting on Merlin, was possible in vestibular schwannoma with a prediction of the postoperative clinical course, it would then be possible for the surgeon to make quick intraoperative decisions, which would benefit the patient (Figure 5).

Another feasible way to examine tumors has been developed. Recently, somatic mutations of tumors were analyzed in the genes of circulating tumor DNA (ctDNA) found in the blood without an open biopsy. This new technology has been used to treat brain tumors [149]. Combining this novel way, “liquid biopsy” with the unveiling of molecular mechanisms of sporadic vestibular schwannoma, it will be possible to determine the tumor’s clinical characteristics by peripheral blood sampling.

This liquid biopsy is now possible by cerebrospinal fluid sampling from patients with vestibular schwannoma [150,151,152]. For example, Huang et al. reported that ATP-binding cassette subfamily A member 3 (ABCA3), secretogranin-1 (SCG1), Krueppel-like factor 11 (KLF11), voltage-dependent calcium channel subunit alpha-2/delta-1 (CA2D1), brain acid soluble protein 1 (BASP1), and peroxiredoxin-2 (PRDX2) in cerebrospinal fluid sampling were associated with vestibular schwannoma growth [151].

If a liquid biopsy is possible more easily and broadly, surgeons can propose early surgery to avoid irreversible hearing loss in high-risk patients and avoid unfortunate hearing loss due to an inappropriate wait and scan. To develop these ideal decision-making methods, the molecular mechanisms of sporadic vestibular schwannoma should be clarified.

## 12. Future Direction

**Figure 6:**
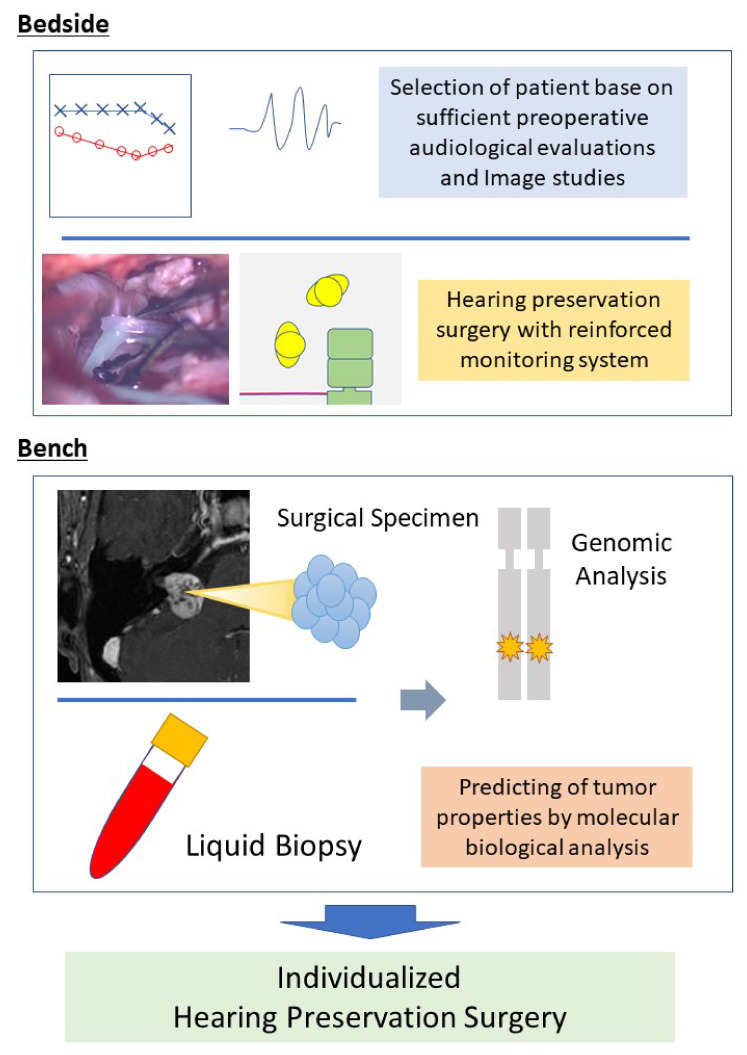
Future directions of hearing preservation surgery combining bedside and bench. The quality of hearing preservation surgery for vestibular schwannoma can be improved by combining sophisticated clinical techniques and molecular analysis of the tumor. In the future, this combination will enable individualized hearing preservation surgery. Further investigation in this field is required.

In this manuscript, we reviewed the recent advances in vestibular schwannoma treatment for hearing preservation from the viewpoint of otologists. We also summarized the latest understanding of the molecular biological mechanism of sporadic vestibular schwannoma. Combining understanding of the clinical viewpoint (Bedside) and molecular biological viewpoint (Bench) of vestibular schwannoma, a new treatment strategy is suggested (Figure 6). This new strategy is not a complete resection surgery for vestibular schwannoma cases but an individualized approach in surgery for hearing preservation based on preoperative residual hearing and the genomic information of the tumor. Residual hearing function in this strategy includes the results of OAE and ABR, as well as pure tone audiometry and word recognition tests. In addition, genomic testing can provide information through rapid intraoperative testing or a preoperative liquid biopsy.

To realize this personalized approach to surgery, in which the patients maintain their hearing ability after surgery while controlling local tumor growth, it is necessary to continue refining intraoperative monitoring technology and to further elucidate the molecular biological mechanism of vestibular schwannoma.

## Data Availability

Not applicable.
